# The Analgesic Enhancing Effects of Coupling M1 and PMC rTMS on Neuropathic Pain After Spinal Cord Injury: An fNIRS Study

**DOI:** 10.1155/prm/4002703

**Published:** 2026-01-30

**Authors:** Xiangbo Wu, Mulan Xu, Wei Sun, Xiaodong Lin, Baijie Xue, Fen Ju, Tao Han, Xinyu Liu, Chenguang Zhao, Xiaolong Sun, Hua Yuan

**Affiliations:** ^1^ Department of Rehabilitation Medicine, Xijing Hospital, Air Force Medical University (Fourth Military Medical University), Xi’an, China, fmmu.edu.cn; ^2^ Department of Rehabilitation Medicine, Sun Yat-Sen Memorial Hospital (Shenshan Medical Center), Sun Yat-sen University, Shanwei, Guangdong, China, sysu.edu.cn

**Keywords:** functional near-infrared spectroscopy, neuropathic pain, premotor cortex, primary motor cortex, repetitive transcranial magnetic stimulation, spinal cord injury

## Abstract

**Background:**

Repetitive transcranial magnetic stimulation (rTMS) of the left primary motor cortex (M1) shows promise for treating neuropathic pain (NP) after spinal cord injury (SCI), but its efficacy remains limited. This study investigated whether combining rTMS on M1 with premotor cortex (PMC) could improve pain relief in SCI patients with NP.

**Materials and Methods:**

Thirty‐nine subjects with NP post‐SCI were randomly assigned to three groups: M1 + PMC (10‐Hz rTMS on left M1 and PMC), M1 (10‐Hz rTMS on left M1), and sham. They underwent daily rTMS sessions for 4 weeks with 2 days off each week. Pain was assessed using the numerical rating scale (NRS) and the Short‐Form McGill Pain Questionnaire‐2 (SF‐MPQ2). Functional near‐infrared spectroscopy (fNIRS) measured activations in bilateral M1, PMC, and primary somatosensory cortex (S1) during a handgrip task.

**Results:**

Pain intensity gradually declined in the M1 + PMC, M1, and sham groups over time. Both the M1 and M1 + PMC groups experienced greater reductions in NRS scores compared to the sham group (*p* < 0.05), with the M1 + PMC group showing the most significant reduction (*p* < 0.05). The M1 + PMC group showed pain relief from Weeks 1 to 6, along with notable inhibition of left M1 and the left PMC activation. The decrease in the oxyhemoglobin (HbO) concentration in the left PMC is significantly positively correlated with the improvement of the NRS score (*r* = 0.607, *p* = 0.028) and SF‐MPQ2 (*r* = 0.595, *p* = 0.032), respectively.

**Conclusions:**

High‐frequency rTMS targeting both left M1 and the left PMC is more effective for NP after SCI than targeting left M1 alone, indicating a synergistic benefit.

**Trial Registration:** Chinese Clinical Trials Registry: ChiCTR2000029024

## 1. Introduction

Spinal cord injury (SCI) stands as a severe neurological trauma, with an annual incidence rate ranging from 8 to 246 cases per million people globally [1]. Among the myriad complications that afflict SCI patients, neuropathic pain (NP) represents a prevalent issue, affecting approximately 50% of this patient population [2]. Characterized by intense and enduring pain, NP can persist for months or even years, significantly compromising the physical and mental well‐being of affected individuals [3]. Addressing NP symptoms is paramount for enhancing the quality of life for these patients. Unfortunately, conventional pharmacological interventions exhibit limited efficacy, delivering suboptimal pain relief and sometimes accompanied by undesirable side effects [4].

Growing evidence implicates cortical overexcitation due to peripheral and central sensitization in the genesis of NP. Consequently, interventions targeting cortical excitability have become pivotal in NP management [5]. Repetitive transcranial magnetic stimulation (rTMS) has become a research hotspot in neuroscience for its noninvasive neuroregulatory capabilities, allowing precise stimulation of brain regions and modulation of neuronal activity [6]. Research indicates that high‐frequency rTMS applied to the primary motor cortex (M1) can restore impaired intracortical inhibition, exhibiting a positive correlation with pain relief [7]. Additionally, rTMS achieves analgesic effects by modulating the balance of inhibitory and excitatory neurotransmitters in the cortex [8]. Recent clinical guidelines endorse the application of high‐frequency rTMS on contralateral M1 as a Grade A recommendation for NP treatment [9]. However, current guidelines do not provide a recommendation grade for NP following SCI [9, 10]. Our prior findings indicate that rTMS targeting M1 necessitates a minimum duration of 2 weeks to elicit significant analgesic effects. Notably, the enhanced pain‐relieving efficacy of high‐frequency rTMS applied to left M1 was temporally associated with reduced neural hypersensitivity in both the stimulated M1 and the ipsilateral premotor cortex (PMC) [5]. Interestingly, a recent study comparing electrical stimulation in PMC and M1 for chronic NP revealed that subdural electrical stimulation in PMC could effectively alleviate NP symptoms, at least as effectively as stimulation in M1 [11]. PMC’s involvement in warm pain threshold regulation, dopamine release from the striatum in pain modulation, and cerebral hemodynamic changes across broader cortical and subcortical regions related to pain modulation suggests its potential significance in NP management [12]. Besides, the interaction between PMC and M1 is thought to enhance intracortical inhibition [13]. Based on these findings, we hypothesized that high‐frequency rTMS targeting both M1 and PMC may be more efficient on pain relief than targeting M1 alone.

Recently, functional near‐infrared spectroscopy (fNIRS) has become a popular noninvasive method in research fields like analgesia [14]. fNIRS reportedly facilitates the real‐time detection of cortical oxyhemoglobin (HbO), with changes in the HbO concentration closely associated with the brain’s activation state [5]. Relative to other neuroimaging modalities such as functional magnetic resonance imaging (fMRI), fNIRS offers enhanced mobility and is less restrictive in terms of movement and susceptibility to electromagnetic interference [15, 16] and therefore is particularly suitable for SCI patients.

## 2. Materials and Methods

### 2.1. Study Participants

This randomized trial enrolled right‐handed inpatients with NP after SCI from Xijing Hospital, Air Force Medical University, between June 2021 and September 2022. The inclusion criteria were as follows: (1) 18–70 years of age; (2) complete or incomplete SCI; (3) presence of NP at or below the lesion level, with moderate to severe pain intensity (≥ 4/10 on the numerical rating scale [NRS]) [5]. Diagnostic criteria for NP were based on the standards recommended by the International Association for the Study of Pain in 2016 [17]; (4) motor evoked potential (MEP) recordable from the first dorsal interosseous (FDI) muscle of the right hand; (5) basic reading and communication skills, enabling the completion of questionnaires and assessment scales.

The exclusion criteria comprised the following: (1) identification of contraindications associated with rTMS, including a history of epilepsy, implanted medical devices, or metallic implants in the head region, as assessed by the Transcranial Magnetic Stimulation Adult Safety Screen questionnaire [18]; (2) personal or family history of psychiatric disorders in first‐degree relatives; (3) severe coexisting systemic diseases, such as cancer, neurologic, or mental disorders; (4) previous rTMS treatment; and (5) pain not of neuropathic origin, including dull or aching pain associated with movement and tenderness of musculoskeletal structures upon palpation. Moreover, during the enrollment phase, patients with NP suspected to be unrelated to SCI, such as those with mononeuropathy, postherpetic neuralgia, and central poststroke pain, were excluded [19].

Patients were permitted to continue their pre‐existing analgesic regimens throughout the study period. The types and distribution of analgesic medications were comparable across the three groups at baseline, as detailed in Supporting Table 1. No new analgesics were initiated during the trial.

This study was approved by the Institutional Review Board (No. KY20192049‐F‐2). Informed consent was obtained from all participants.

### 2.2. Sample Size

The determination of the sample size was based on prior clinical studies investigating the analgesic effects of rTMS on NP [5]. High‐frequency rTMS on M1 has demonstrated an efficacy of approximately 65% in pain alleviation, whereas conventional treatment yields a pain relief rate of around 10% [10]. Assuming a 70% pain relief rate with high‐frequency rTMS on M1/PMC, power analysis (G^∗^Power, *α* = 0.05, power = 80%) indicated 11 subjects per group.

### 2.3. Study Design

Participants were randomly assigned to one of three groups in a 1:1:1 ratio using a random number table: M1 + PMC group (received rTMS on left M1 followed by the left PMC), M1 group (received rTMS on left M1 and sham stimulation on the left PMC), and sham group (received sham stimulation on left M1 and the left PMC). All patients received rTMS or sham stimulation once a day, five times per week, for 4 weeks. rTMS was performed before routine rehabilitation treatment. Routine rehabilitation interventions and medication treatments (including analgesics) were not altered during the entire experimental process.

The NRS and the Short‐Form McGill Pain Questionnaire‐2 (SF‐MPQ2) were evaluated at baseline (T0), after the first rTMS or sham stimulation session (Day 1, T1), and at the end of week 1 (T2), 2 (T3), 4 (T4), and 6 (T5). FNIRS tests were conducted at baseline (T0), Week 2 (T3), and Week 4 (T4) (Supporting Figure 1). The participants, evaluators, and data analysts were blinded throughout the study.

### 2.4. rTMS Stimulation Intervention

A figure‐of‐eight magnetic coil connected to the CCY‐1 stimulator (YIRUIDE Medical Equipment Company, Wuhan, China) was utilized for rTMS delivery. Initially, the coil was positioned on left M1 and then carefully adjusted to its optimal location, eliciting the maximum MEP in the right FDI muscle, identified as the “hot spot.” Resting motor threshold (RMT) was defined as the minimum stimulus intensity causing MEP ≥ 50 μV in at least 5 out of 10 consecutive stimuli [20]. PMC stimulation was defined as 2 cm anterior and 1 cm medial from FDI activation hotspots previously defined [13]. Trained therapists with over 3 years of experience in rTMS treatment conducted the rTMS sessions. The figure‐of‐eight magnetic coil was positioned on PMC or M1 as required [1]. The coil for the sham stimulation group was positioned on the scalp at a 90° angle, maintaining the same site and stimulation parameters as those used for the real rTMS group [21].

The M1 group received 10‐Hz rTMS over left M1 (80% RMT, 1000 pulses, 1‐s trains, 3‐s intervals) [5, 22]. Sham stimulation over the left PMC used a 90° coil angle to mimic 10‐Hz auditory effects.

For the M1 + PMC group, administer 10‐Hz rTMS to left M1 at 80% of the RMT, delivering a total of 1000 pulses. Each train should have a duration of 1 s, followed by a 3‐s intertrain interval. Subsequently, apply 10‐Hz rTMS to the left PMC, also delivering a total of 1000 pulses, with each stimulus lasting 1 s and a 3‐s interval between stimuli.

For the sham group, simulated stimulation was administered to left M1 using a coil positioned at a 90‐degree angle to the scalp, thereby replicating the auditory effects associated with 10‐Hz stimulation. Subsequently, simulated stimulation was similarly applied to the left PMC, with the coil again positioned at a 90‐degree angle to the scalp, to reproduce the noise characteristic of 10‐Hz stimulation.

### 2.5. fNIRS Acquisition

The fNIRS system (NirSmart‐6000A, Danyang Huichuang Medical Equipment Co., Ltd., Jiangsu, China) detected near‐infrared light absorption. The optical channel locations and probe montage were determined based on the international 10–20‐electrode system, focusing on regions of interest (ROIs) such as bilateral M1, primary somatosensory cortex (S1), and PMC (Figure 1(a)). Using the modified Beer–Lambert law, optical data were converted into changes in the HbO concentration to analyze the activation of corresponding brain regions [23].

FIGURE 1Experimental setup. (a) Layout of the fNIRS probe array. The fNIRS system includes eight detectors (red) and eight sources (blue), located according to the international 10–20 systems. ROIs are represented by the dashed lines, and the corresponding anatomical positions of each channel are shown in the table. (b) The experimental paradigm consists of three conditions, namely, resting state, left handgrip task, and right handgrip task. Abbreviations: ROIs: regions of interest; fNIRS: functional near‐infrared spectroscopy; LPMC, left premotor cortex; RPMC, right premotor cortex; LM1, left primary motor cortex; RM1, right primary motor cortex; LS1, left primary somatosensory cortex; RS1, right primary somatosensory cortex.

**Figure:**
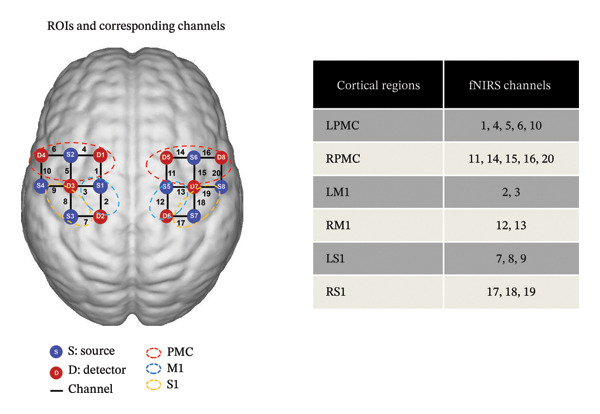
(a)

**Figure:**
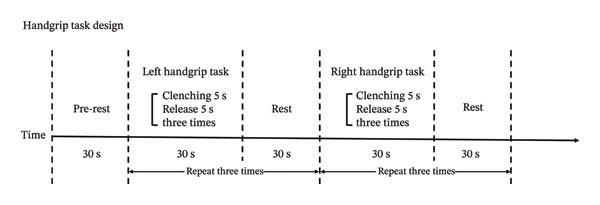
(b)

During the handgrip task, fNIRS data were recorded following a modified protocol from our previous study [5]. The task included (1) a 30‐s rest; (2) left task, clenching fists for 5 s, followed by release for 5 s, repeated three times; (3) a 30‐s rest, with steps two to three repeated three times; (4) right task, clenching fists for 5 s, followed by release for 5 s, repeated three times; and (5) a 30‐s rest, with steps four to five repeated three times (Figure 1(b)).

### 2.6. Outcome Measurements—NRS and SF‐MPQ2

The primary assessment criterion for evaluating the efficacy of this study was the analgesic effect observed in patients after treatment. To quantitatively measure this effect, both the NRS scale and SF‐MPQ2 scale were employed. The NRS scale facilitates the quantitative evaluation of subjects’ pain intensity, utilizing a numerical range from 0 to 10, making it one of the most widely utilized pain assessment scales in both clinical and scientific research contexts [24]. The treatment response rate was compared among the three groups based on two criteria: (1) pain relief rate, where, at the end of treatment, the NRS score decreased by more than 30% compared to the baseline, and (2) significant pain relief rate, defined as a reduction of more than 50% in the NRS score compared to the baseline [10].

The SF‐MPQ2 serves as a comprehensive, multi‐dimensional pain assessment scale, offering a quantitative evaluation of various pain attributes, including type, intensity, and emotional perception [25]. This questionnaire is structured into four primary dimensions: persistent pain, paroxysmal pain, NP, and the impact on emotion, encompassing a total of 22 subitems. Higher scores indicate more severe symptoms. Renowned for its high reliability and validity, this scale is extensively used in clinical settings [26].

### 2.7. fNIRS Data Processing

The fNIRS data analysis focused on the HbO concentration, the most sensitive indicator of cerebral blood flow dynamics [5]. Data were processed using the NirSpark analysis software. Preprocessing steps included automatic motion artifact correction and band‐pass filtering (0.01–0.2 Hz) to remove physiological noise [27]. Considering the hemodynamic response characteristics of the handshake task, which exhibits a 3‐s lag and reaches its peak in the fifth second [28], the blockavg module was employed to calculate the average HbO concentration during the clenched fist period (3–7 s). The hemodynamic response during the handgrip task was modeled, and the average HbO concentration during the active task period (3–7 s after movement onset) was calculated for each channel and then averaged across the nine task blocks [29]. For statistical analysis, the mean HbO values from channels corresponding to each ROI (bilateral M1, PMC, and S1) were used [30].

### 2.8. Statistical Analysis

Statistical analysis was performed utilizing SPSS Version 24.0. Categorical data were expressed as frequencies and percentages, and intergroup comparisons were conducted using Fisher’s exact test. For measurement data, normality, and variance homogeneity tests were performed. For normally distributed measurement data, results were expressed as mean ± standard error of mean (SEM). Analysis of variance (ANOVA) was utilized to compare the differences at baseline for each group. Paired *t* tests were conducted to compare HbO concentration differences between left and right brain regions. Non‐normally distributed data were presented as median (M) and interquartile range (IQR). Intragroup comparisons were performed using the Wilcoxon paired rank sum test, and intergroup comparisons used the Kruskal–Wallis *H* test. To evaluate the impact of various intervention measures on the NRS and SF‐MPQ2, a repeated measures ANOVA was conducted. Additionally, the cumulative effect of these interventions on changes in the HbO concentration was assessed using repeated measures ANOVA, with pairwise comparisons performed using the least significant difference *t* test (LSD‐t). Perform correlation analysis using Spearman. *p* < 0.05 was considered statistically significant for all analyses, including post hoc LSD tests.

## 3. Results

### 3.1. Study Flowchart and Baseline Data of Patients

**FIGURE 2:**
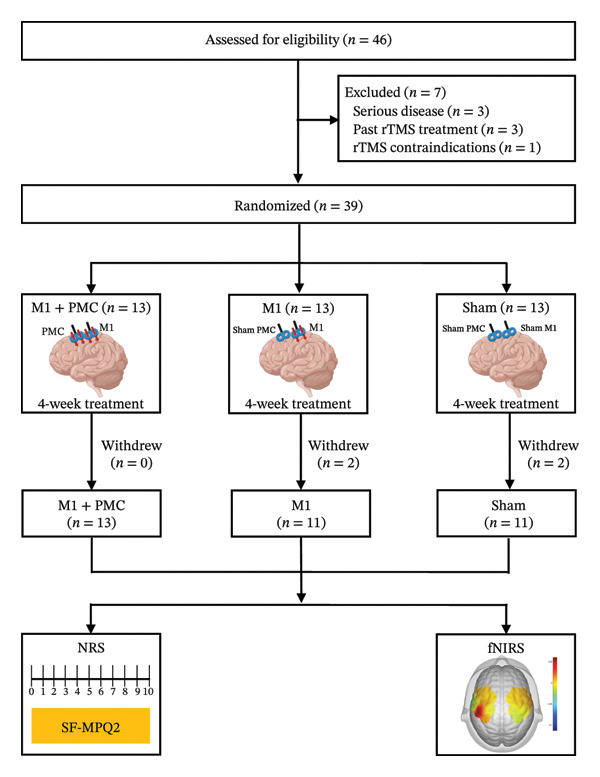
Treatment study flowchart. Abbreviations: rTMS: repetitive transcranial magnetic stimulation; M1: motor cortex; PMC: premotor cortex; NRS: numerical rating scale; SF‐MPQ2: the Short‐Form McGill Pain Questionnaire‐2; fNIRS: functional near‐infrared spectroscopy.

A total of 39 eligible patients were randomly assigned to the M1 + PMC group (*n* = 13), M1 group (*n* = 13), and the sham group (*n* = 13). Two patients in the M1 group and two patients in the sham group withdrew due to personal reasons, resulting in 35 subjects completing the treatment and being included in the final analysis (Figure 2). There were no significant differences in demographic, baseline characteristics, and analgesic treatments across the three groups (Table 1 and Supporting Table 1).

**TABLE 1:** Comparison of baseline data for the study population included in three groups.

Characteristics	M1 + PMC (*n* = 13)	M1 (*n* = 11)	Sham (*n* = 11)	*x* ^2^/F/Z	*p*
Gender					
Male	9 (69.2%)	10 (90.9%)	7 (63.6%)	2.436	0.383^a^
Female	4 (30.8%)	1 (9.1%)	4 (36.4%)		
Age, y	46.9 ± 3.2	44.6 ± 3.8	41.8 ± 3.4	0.537	0.590^b^
Education					
Low	5 (38.5%)	6 (54.5%)	5 (45.5%)	2.007	0.798^a^
Medium	5 (38.5%)	3 (27.3%)	2 (18.2%)		
High	3 (23.1%)	2 (18.2%)	4 (36.4%)		
Career					
Manual	8 (61.5%)	9 (81.8%)	5 (45.5%)	3.048	0.233^a^
Other	5 (38.5%)	2 (18.2%)	6 (54.5%)		
Cause of injury					
Heavy pound	0 (0.0%)	3 (27.3%)	0 (0.0%)	9.763	0.089^a^
Traffic accidents	4 (30.8%)	0 (0.0%)	2 (18.2%)		
Fall	5 (38.5%)	2 (18.2%)	5 (45.5%)		
Other	4 (30.8%)	6 (54.5%)	4 (36.4%)		
Injury level					
Cervical	6 (46.2%)	3 (27.3%)	2 (18.2%)	3.059	0.606^a^
Thoracic	5 (38.5%)	4 (36.4%)	6 (54.5%)		
Lumbar‐sacral	2 (15.4%)	4 (36.4%)	3 (27.3%)		
Injury degree					
Complete	3 (23.1%)	4 (36.4%)	5 (45.5%)	1.403	0.552^a^
Incomplete	10 (76.9%)	7 (63.6%)	6 (54.5%)		
Duration, median (IQR), m	2.0 (1.0, 4.0)	2.0 (1.0, 10.0)	3.0 (1.0, 4.0)	0.380	0.827^c^
Type of NP					
At level pain	1 (7.7%)	3 (27.3%)	2 (18.2%)	3.158	0.566^a^
Below level pain	7 (53.8%)	4 (36.4%)	7 (63.6%)		
Both	5 (38.5%)	4 (36.4%)	2 (18.2%)		
Analgesics					
Yes	9 (69.2%)	10 (90.9%)	9 (81.8%)	1.672	0.506^a^
No	4 (30.8%)	1 (9.1%)	2 (18.2%)		
Baseline NRS	5.5 ± 0.2	5.5 ± 0.2	5.6 ± 0.2	0.045	0.957^b^
Baseline SF‐MPQ2	21.2 ± 2.2	21.6 ± 2.1	21.4 ± 3.0	0.007	0.993^b^

*Note:* M1: motor cortex; PMC: premotor cortex; IQR: interquartile range; m: month; SF‐MPQ2: the simplified Chinese version of McGill Pain Questionnaire‐2.

^a^Fisher’s exact test.

^b^ANOVA.

^c^Kruskal–Wallis test.

### 3.2. NRS and SF‐MPQ2 Response

The time cumulative effect of different interventions on NRS scores for the three groups was analyzed using a two‐way repeated measures ANOVA, with post hoc analysis revealing a gradual decrease in NRS scores over time (Figure 3(a)). Both the main effects of time and group were significant (*p* < 0.05), and post hoc analysis indicated significant differences among the three groups. A significant interaction between time and group was observed (*p* < 0.05), prompting simple effects analysis. The results showed significant effects of both time and group (*p* < 0.05). Notably, the M1 + PMC group exhibited a significant difference from the sham group starting from Week 1, and the M1 group showed significant differences from the sham group starting from Week 2, with the M1 + PMC group differing significantly from the M1 group (*p* < 0.05).

FIGURE 3Changes in pain scores over time. (a) Changes in the NRS scores over time. (b) Changes in the SF‐MQP2 scores over time. ^∗^ *p* < 0.05 between the M1 + PMC group and sham group. ^#^ *p* < 0.05 between the M1 group and sham group. ^+^ *p* < 0.05 between the M1 group and M1 + PMC group. Abbreviations: M1: motor cortex; PMC: premotor cortex; NRS: numerical rating scale; SF‐MPQ2: the Short‐Form McGill Pain Questionnaire‐2; w: week.

**Figure:**
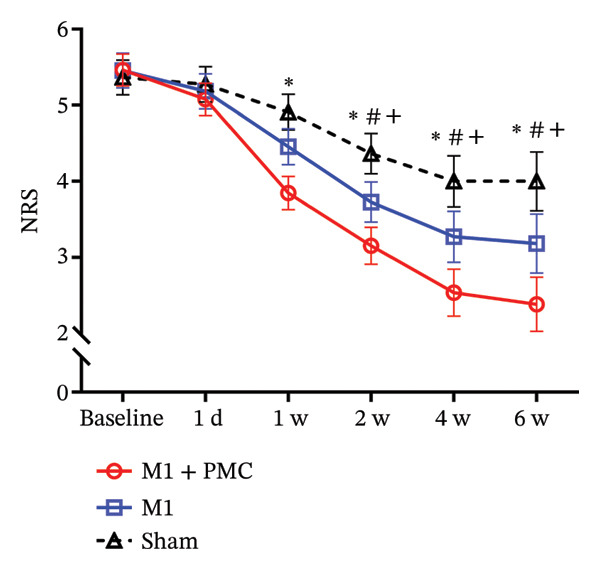
(a)

**Figure:**
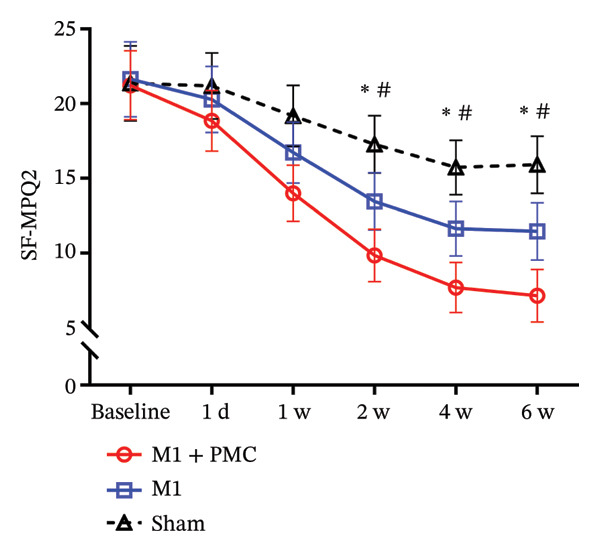
(b)

Similarly, a two‐way repeated measures ANOVA for SF‐MPQ2 scores revealed a significant difference among the three groups (*p* < 0.001, Figure 3(b)). Post hoc analysis demonstrated significant differences between the M1 + PMC group and sham group (*p* < 0.001), as well as between the M1 group and sham group (*p* < 0.05). The decrease in SF‐MPQ2 scores for the M1 + PMC group and M1 group was greater than that for the sham group. Although the decrease in SF‐MPQ2 scores for the M1 + PMC group was higher than that for the M1 group, the difference did not reach statistical significance (*p* = 0.061). An interaction between time and group was also observed (*p* < 0.05), and simple effects analysis revealed significant effects of both time and group (*p* < 0.05). The M1 + PMC group showed a significant difference from the sham group starting at 2 weeks, and the M1 group differed significantly from the sham group at the same time point (*p* < 0.05).

### 3.3. Pain Relief

After 4 weeks of treatment, the pain relief rates, based on a threshold of 30% improvement (R% > 30%), were higher in both the M1 + PMC group (76.9%) and the M1 group (72.7%) compared to the sham group (36.4%). However, the difference in pain relief rates among the three groups did not reach statistical significance (*p* = 0.088) (Table 2).

**TABLE 2:** Comparison of pain relief and significant pain relief of the three groups after treatment.

*R* (%)	M1 + PMC	M1	Sham	*x* ^2^	*p*
> 30	10 (76.9%)	8 (72.7%)	4 (36.4%)	4.868	0.088
≤ 30	3 (23.1%)	3 (27.3%)	7 (63.6%)		

> 50	6 (46.2%)	3 (27.3%)	0 (0.0%)	6.665	0.036
≤ 50	7 (53.8%)^a^	8 (72.7%)^a^	11 (100.0%)		

*Note:* M1: motor cortex; PMC: premotor cortex; *R* (%): pain relief.

^a^ *p* < 0.05, compared with the sham group.

When considering the significant pain relief rate (R% > 50%), the M1 + PMC group (46.2%) and M1 group (27.3%) showed higher rates than the sham group (0%), and the difference among the three groups was statistically significant (*p* = 0.036). Post hoc pairwise comparisons revealed that the difference between the M1 + PMC group and the M1 group did not reach statistical significance (*p* = 0.423, Table 2).

### 3.4. fNIRS Test Results

At baseline, there was no significant difference in the average HbO concentration among the three groups (Supporting Figure 2). During the left handgrip task, no difference in the average HbO concentration was found for LPMC vs. RPMC, LM1 vs. RM1, and LS1 vs. RS1 among the three groups before rTMS or sham treatment (Figures 4(a), 4(b)). During the right handgrip task, the average HbO concentration of LPMC and LM1 in the three groups was significantly higher than that in the corresponding brain cortices on the right side (RPMC and RM1), but there was no significant difference in LS1 and RS1 (*p* > 0.05, Figures 4(a), 4(c)).

FIGURE 4(a) HbO activation maps for the left and right handgrip tasks in the M1 + PMC, M1, and sham groups before rTMS treatment. The color scale represents the intensity of HbO activation, with warmer colors (e.g., red) indicating higher activation and cooler colors (e.g., blue) indicating lower activation. (b) Before rTMS treatment, the comparison of the average HbO concentrations of each ROI on the left and right sides during the left handgrip task in the M1 + PMC group, M1 group, and sham group. (c) Before rTMS treatment, the comparison of the average HbO concentrations of each ROI on the left and right sides during the right handgrip task in the M1 + PMC group, M1 group, and sham group. ^∗^ *p* < 0.05. Abbreviations: M1: motor cortex; PMC: premotor cortex; S1: primary somatosensory cortex.

**Figure:**
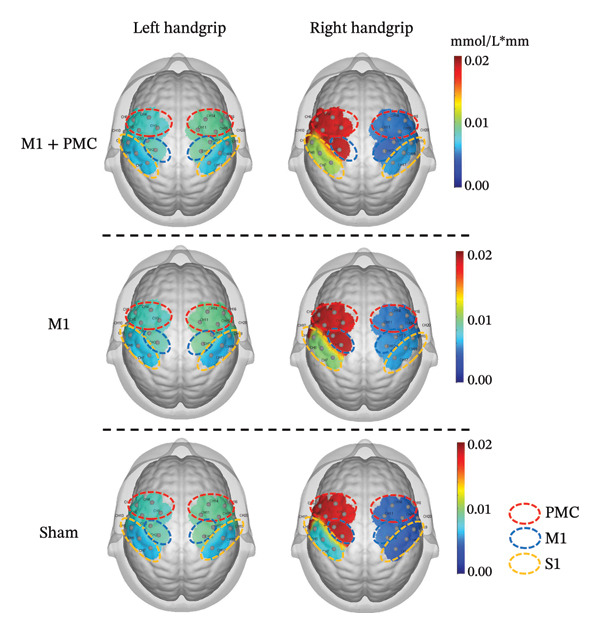
(a)

**Figure:**
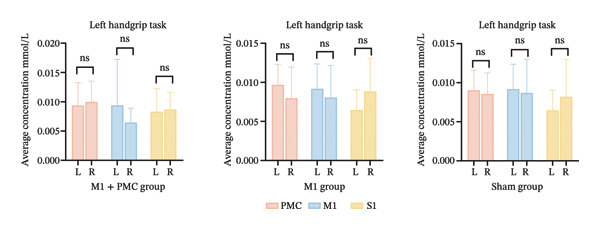
(b)

**Figure:**
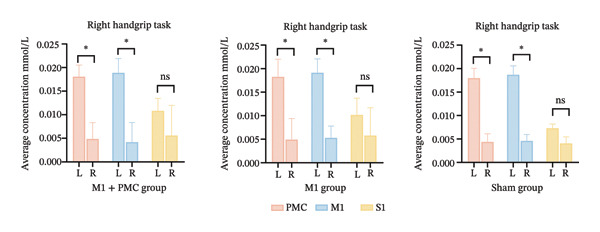
(c)

After 2 weeks of treatment, the average HbO concentration in LPMC and LM1 of the M1 + PMC group and M1 group was significantly lower than the sham group, and this difference persisted until 4 weeks after the first treatment (*p* < 0.05). However, the average HbO concentration in LPMC and LM1 of the M1 + PMC group and M1 group was not significantly different (*p* > 0.05, Figure 5).

FIGURE 5(a) HbO activation maps for the right handgrip task in the M1 + PMC group, M1 group, M1 group, and sham group at baseline, 2 weeks of treatment, and 4 weeks of treatment. The color scale represents the intensity of HbO activation, with warmer colors (e.g., red) indicating higher activation and cooler colors (e.g., blue) indicating lower activation. (b) Comparing the average HbO concentrations of LPMC in each group at three time points. (c) Comparing the average HbO concentrations of LM1 in each group at three time points. ^∗^ *p* < 0.05. Abbreviations: M1: motor cortex; PMC: premotor cortex; S1: primary somatosensory cortex; LPMC, left premotor cortex; LM1, left primary motor cortex.

**Figure:**
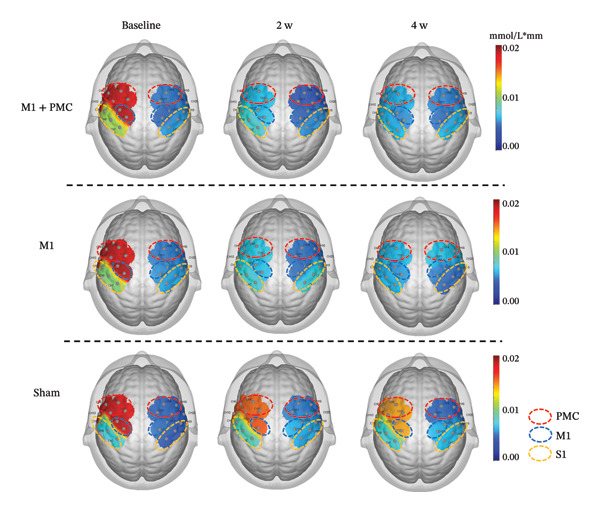
(a)

**Figure:**
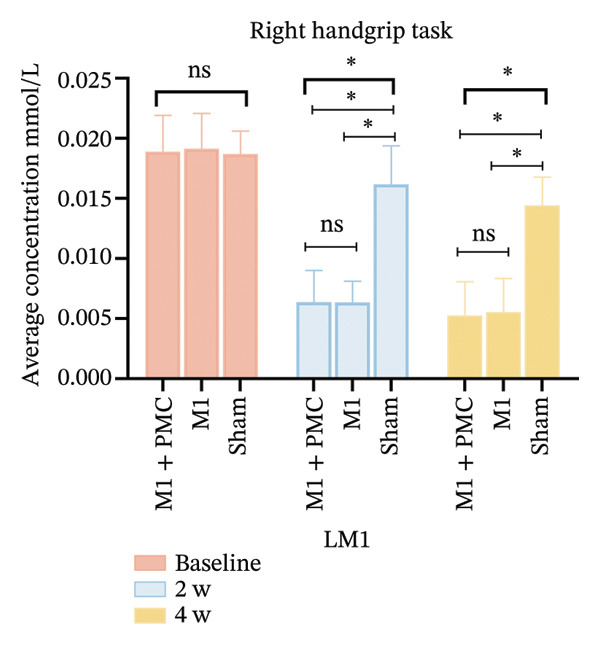
(b)

**Figure:**
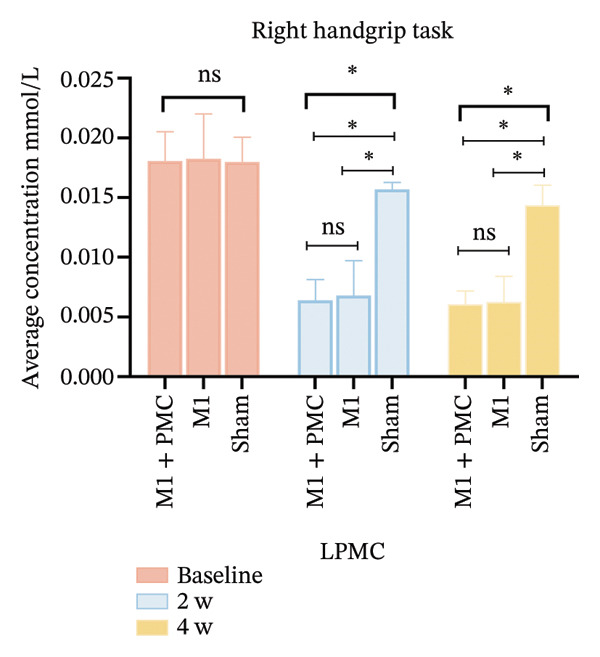
(c)

Figure 6 illustrates the relationship between the reduction in HbO levels in the LPMC and the improvement in pain scores within the M1 + PMC group at 2 weeks. The decrease in the HbO concentration in the LPMC is significantly positively correlated with the enhancement of the NRS score (*r* = 0.607, *p* = 0.028) and the SF‐MPQ2 score (*r* = 0.595, *p* = 0.032), as depicted in Figures 6(a) and 6(b), respectively.

FIGURE 6The correlation between the decrease in HbO concentrations in LPMC and the improvement of pain score at 2 weeks in the M1 + PMC group. (a) The correlation between the decrease of HbO in LPMC and the improvement of the NRS score. (b) The correlation between the decrease of HbO in LPMC and the improvement of SF‐MPQ2. Abbreviations: NRS: numerical rating scale; SF‐MPQ2: the Short‐Form McGill Pain Questionnaire‐2.

**Figure:**
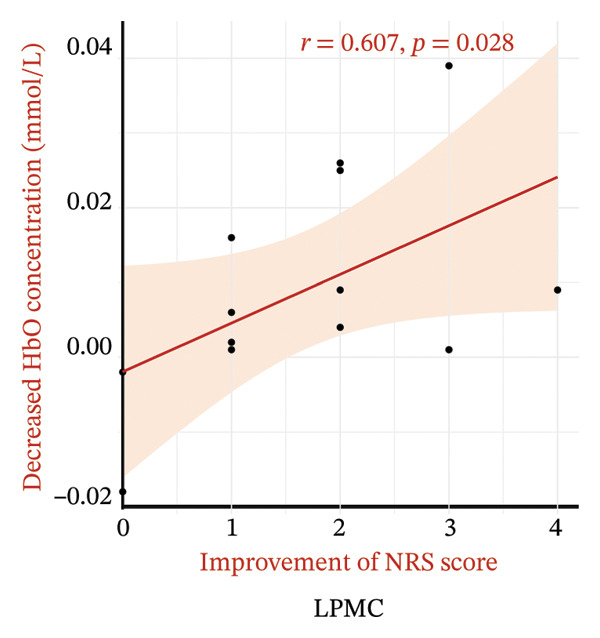
(a)

**Figure:**
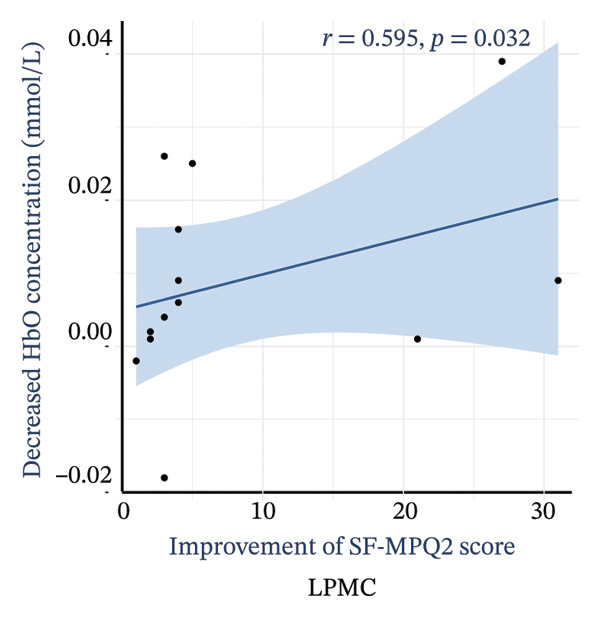
(b)

### 3.5. Blinding Assessment

At the end of the study, 17 out of 35 patients were able to report which group they were allocated to, and 10 out of 17 patients were correct (58.82%). The chi‐square test showed that there was no significant difference in the proportion of correct rates of group awareness among the three groups (*p* = 0.789, Supporting Table 2).

## 4. Discussion

This study substantiated that, compared to the stimulation of M1 alone, high‐frequency rTMS applied to M1 combined with the PMC confers significant benefits in alleviating NP. Simultaneously, the study revealed a significant inhibition of motor‐related regions, including M1 and PMC, during handgrip tasks.

This study found that while single‐session rTMS showed limited analgesia, repeated stimulation produced cumulative effects, with the M1 + PMC combination demonstrating superior outcomes (46.2% pain relief rate) compared to M1‐alone (27.3%) or sham (0%), suggesting that dual‐target stimulation more effectively alleviates NP. While several studies have reported the efficacy of rTMS on M1 in alleviating average and severe NP after SCI [31, 32] some have indicated that M1 stimulation alone may yield no response or only temporary effects, with an overall effective rate of approximately 40% [10]. This has led researchers to explore alternative stimulation targets, including the dorsolateral prefrontal cortex (DLPFC), PMC, frontal cortex, and secondary somatosensory cortex (S2) [33]. Recent studies indicate that the PMC, in conjunction with the M1 cortex, is organized into a neuronal network responsible for the intricate control of movements and sensorimotor integration [11]. The specific mechanisms underlying the organization and functioning of this network require further investigation, which may advance the development of novel strategies for the treatment of NP. Abnormal pain stimuli have been shown to significantly increase the activation of M1 and PMC [5]. During the handgrip task, this study observed significant activation of PMC and M1, which, after multiple rTMS treatments, was significantly inhibited, concomitant with a decrease in the pain intensity [5]. A study comparing the efficacy of electrical stimulation in the PMC and M1 for treating chronic NP found that subdural electrical stimulation in the PMC could significantly control NP symptoms, with effects comparable to those in M1, especially for refractory NP [11]. The current study corroborated these findings, demonstrating that high‐frequency rTMS on M1 combined with PMC outperformed M1 stimulation alone in terms of analgesic effect.

Besides NRS, the study observed a greater decrease in SF‐MPQ2 scores in both the M1 + PMC group and M1 group compared to the sham group, although the difference between the M1 + PMC and M1 groups was not statistically significant (*p* > 0.05). Several potential factors warrant consideration in interpreting these findings. Firstly, SF‐MPQ2 encompasses a comprehensive assessment of pain, encompassing various dimensions and emotional experiences [26]. Secondly, the limited sample size in this study may contribute to the absence of significant differences between the M1 + PMC and M1 groups.

To elucidate the cortical mechanisms underlying the analgesic effects of rTMS, we examined task‐related brain activation using fNIRS. Our key finding was that the superior pain relief in the M1 + PMC group was associated with a significant suppression of activation in the LPMC and LM1 cortices. This observation aligns with the theory of neurovascular coupling and suggests that the therapeutic action of rTMS may involve the normalization of pathological overactivity in this motor‐related network [34]. PMC and M1 are integral components of a neural network responsible for intricate motor control and sensory motor integration [11]. Following treatment, the decrease in the average HbO concentration in PMC and M1 for the M1 + PMC group and M1 group exceeded that in the sham group [5]. This suggests the involvement of the motor cortex in the cumulative analgesic enhancement effect of rTMS treatment. Studies have shown that NP is linked to motor cortex disinhibition, possibly involving an imbalance between γ‐aminobutyrate (GABA) and glutamate in NP patients [35]. High‐frequency rTMS on the motor cortex may enhance GABA synaptic connections, restore the original cortical oscillation frequency, and rectify defective intracortical inhibitory function, thereby producing analgesic effects [36]. Besides, pain sensations are intricately formed in the cortex, with multiple structures in the cerebral cortex and subcortex forming a complex network known as the pain matrix, which is responsible for generating and regulating pain sensations. This matrix comprises three primary functional regions: the lateral neural network encoding sensory information and generating pain stimuli descriptions, the intermediate network primarily involved in encoding emotional aspects of pain, and the pain regulation region encompassing brain regions such as the PFC, M1, PMC, as well as the striatum and cerebellum, associated with motor function [37]. Hence, M1 and PMC are not solely exercise‐related areas but actively participate in pain regulation [38]. It has been established that excessive activity in PMC and M1 after SCI influences the pain intensity by regulating the level of spontaneous neural activity in brain areas related to pain control [8]. M1, PMC, thalamus, and periaqueductal gray matter (PAG) are identified as crucial components of the cerebral pain and motor regulatory network [8]. Given these insights, it is proposed that the analgesic effect of high‐frequency rTMS on M1 and PMC may be linked to activity changes in related brain regions within the cerebral pain and motor regulatory network.

The M1 + PMC group showed a greater NRS reduction than M1 alone, although HbO concentration changes were similar between groups, suggesting that rTMS may alleviate pain by modulating M1/PMC overactivation. Furthermore, other mechanisms may contribute to a superior analgesic effect in the M1 + PMC group including the synergistic effects of multiple targets [39], changes in cortical excitability [8], alterations in functional connectivity within the brain’s pain regulation network [40], and modifications in endogenous opioid receptors [41]. Studies have demonstrated that chronic NP can induce overactivity in thalamic‐related nuclei. Cortical stimulation, particularly in regions with high functional connectivity and plasticity like PMC and M1, may alleviate NP symptoms by suppressing abnormal thalamic discharges [42]. In addition, cortical stimulation can enhance inhibitory GABA activity in the cortex and boost the secretion of endogenous opioids in various structures, primarily in the ACC and PAG, thereby exerting an analgesic effect [43]. Unlike the somatotype distribution with distinct location features in M1, PMC appears to be distributed in a functional pattern. Overlapping areas in PMC represent different movement patterns, potentially indicating complex sensorimotor function control involving multiple body parts and higher cortical plasticity [44]. The regions governing distinct body parts in the PMC may exhibit more dispersed and overlapping traits compared to M1. This phenomenon is associated with the intricate control of sensorimotor functions involving multiple body parts, possibly indicating higher cortical plasticity [45].

This study has several limitations, including its single‐center design and modest sample size, which may affect the generalizability of findings. While we mitigated potential biases through a randomized, sham‐controlled, blinded‐endpoint design and standardized protocols, the reliance on subjective pain scales (NRS/SF‐MPQ2) and variable physiological factors like HbO concentrations may influence results. Additionally, the subgroup analyses—such as those based on disease duration, level of injury, or concomitant use of analgesics—were limited by small sample sizes in certain strata, preventing robust conclusions. Future multicenter trials with larger cohorts are needed to validate these findings, further explore the influence of these patient characteristics on treatment response, and elucidate the underlying mechanisms of rTMS‐mediated analgesia.

## 5. Conclusions

High‐frequency rTMS on M1 + PMC is more effective than on M1 alone, indicating synergistic and cumulative benefits when multiple targets are treated. High‐frequency rTMS on M1 + PMC provides quicker and more significant pain relief. This suggests that high‐frequency rTMS on M1 + PMC could be considered, particularly for refractory NP cases where high‐frequency rTMS on M1 alone is ineffective.

## Author Contributions

Hua Yuan and Xiaolong Sun: designed this manuscript. Xiangbo Wu: writing–review and editing, conceptualization, visualization, and fNIRS detection. Mulan Xu: writing–review and editing, conceptualization, and visualization. Wei Sun and Fen Ju: conducted the statistical analysis. Baijie Xue and Xiaodong Lin: conducted clinical assessments. Tao Han, Xinyu Liu, and Chenguang Zhao: participated in drawing of figures. Xiangbo Wu and Mulan Xu contributed equally to this article. All authors contributed to the article.

## Funding

This work was supported by grants from the National Natural Science Foundation of China (82072534, 82472593, and 82272591) and the Medical Staff Training & Boost Project of Xijing Hospital (XJZT24JC19, XJZT24LY11, and XJZT24QN20).

## Disclosure

All authors read and approved the final manuscript for submission.

## Ethics Statement

All procedures were approved by the Ethics Review Committee of the Xijing Hospital Affiliated to Air Force Medical University (KY20192049‐F‐2).

## Conflicts of Interest

The authors declare no conflicts of interest.

## Supporting Information

Additional supporting information can be found online in the Supporting Information section.

## Supporting information


**Supporting Information 1** Supporting Table 1 shows the analgesic treatment results for spinal cord injury patients.

**Supporting Information 2** Supporting Table 2 presents the results of comparison of correct rate of blinding assessment in three groups.

**Supporting Information 3** Supporting Figure 1 shows the trial timetable. Trial timetable. Pain intensity was evaluated by means of NRS and SF‐MPQ2 at six different time points: T0 (before treatment), T1 (on the first day), T2 (after 1 week), T3 (after 2 weeks), T4 (after 4 weeks), and T5 (after 6 weeks). fNIRS was evaluated only at T0, T3, and T4. Abbreviations: NRS: numerical rating scale; SF‐MPQ2: the Short‐Form McGill Pain Questionnaire‐2; fNIRS: functional near‐infrared spectroscopy.

**Supporting Information 4** Supporting Figure 2 compares the baseline average HbO concentrations in bilateral cortical regions across three groups during both left and right handgrip tasks. (A) Comparison of average HbO concentrations in the left cortex of three groups at baseline for the left handgrip task. (B) Comparison of average HbO concentrations in the right cortex of three groups at baseline for the left handgrip task. (C) Comparison of average HbO concentrations in the left cortex of three groups at baseline for the right handgrip task. (D) Comparison of average HbO concentrations in the right cortex of three groups at baseline for the right handgrip task. Abbreviations: LPMC, left premotor cortex; RPMC, right premotor cortex; LM1, left primary motor cortex; RM1, right primary motor cortex; LS1, left primary somatosensory cortex; RS1, right primary somatosensory cortex.

## Data Availability

The data that support the findings of this study are available from the corresponding author upon reasonable request.
