# Supplementary material for: The Analgesic Enhancing Effects of Coupling M1 and PMC rTMS on Neuropathic Pain After Spinal Cord Injury: An fNIRS Study
Source: Pain Res Manag. 2026 Jan 30;2026:4002703. doi: 10.1155/prm/4002703 (PMC12859385; doi:10.1155/prm/4002703)
Supplement: Supplementary file 1 — Supporting Information 1 Supporting Table 1 shows the analgesic treatment results for spinal cord injury patients. [file PRM-2026-4002703-s001.docx]

| **Supplemental table 1 Drug analgesic treatment for patients with spinal cord injury** | | | |
| --- | --- | --- | --- |
| **Analgesics^a^** | **M1+PMC** | **M1** | **Sham** |
| None | 4 | 1 | 2 |
| Anti-inflammatories | 3 | 1 | 1 |
| Antidepressants | 2 | 1 | 1 |
| Anti-epileptics | 6 | 8 | 8 |
| Benzodiazepines | 1 | 0 | 0 |
| Skeletal muscular relaxants | 0 | 2 | 1 |
| ^a^Some patients were taking more than one. Abbreviations: M1: motor cortex; PMC: premotor cortex. | | | |
