# Supplementary material for: The Analgesic Enhancing Effects of Coupling M1 and PMC rTMS on Neuropathic Pain After Spinal Cord Injury: An fNIRS Study
Source: Pain Res Manag. 2026 Jan 30;2026:4002703. doi: 10.1155/prm/4002703 (PMC12859385; doi:10.1155/prm/4002703)
Supplement: Supplementary file 2 — Supporting Information 2 Supporting Table 2 presents the results of comparison of correct rate of blinding assessment in three groups. [file PRM-2026-4002703-s002.docx]

**Supplemental table 2 Comparison of correct rate of blinding assessment in three groups**

| **Group** | **M1+PMC** | **M1** | **Sham** | **χ^2^** | ***P*** |
| --- | --- | --- | --- | --- | --- |
| Correct rate | 4/13(30.8%) | 3/11(27.3%) | 3/11(27.3%) | 0.475 | 0.789 |

Abbreviations: M1: motor cortex; PMC: premotor cortex.
