# Supplementary figures and images for: The Analgesic Enhancing Effects of Coupling M1 and PMC rTMS on Neuropathic Pain After Spinal Cord Injury: An fNIRS Study
Source: Pain Res Manag. 2026 Jan 30;2026:4002703. doi: 10.1155/prm/4002703 (PMC12859385; doi:10.1155/prm/4002703)

## Slide 1
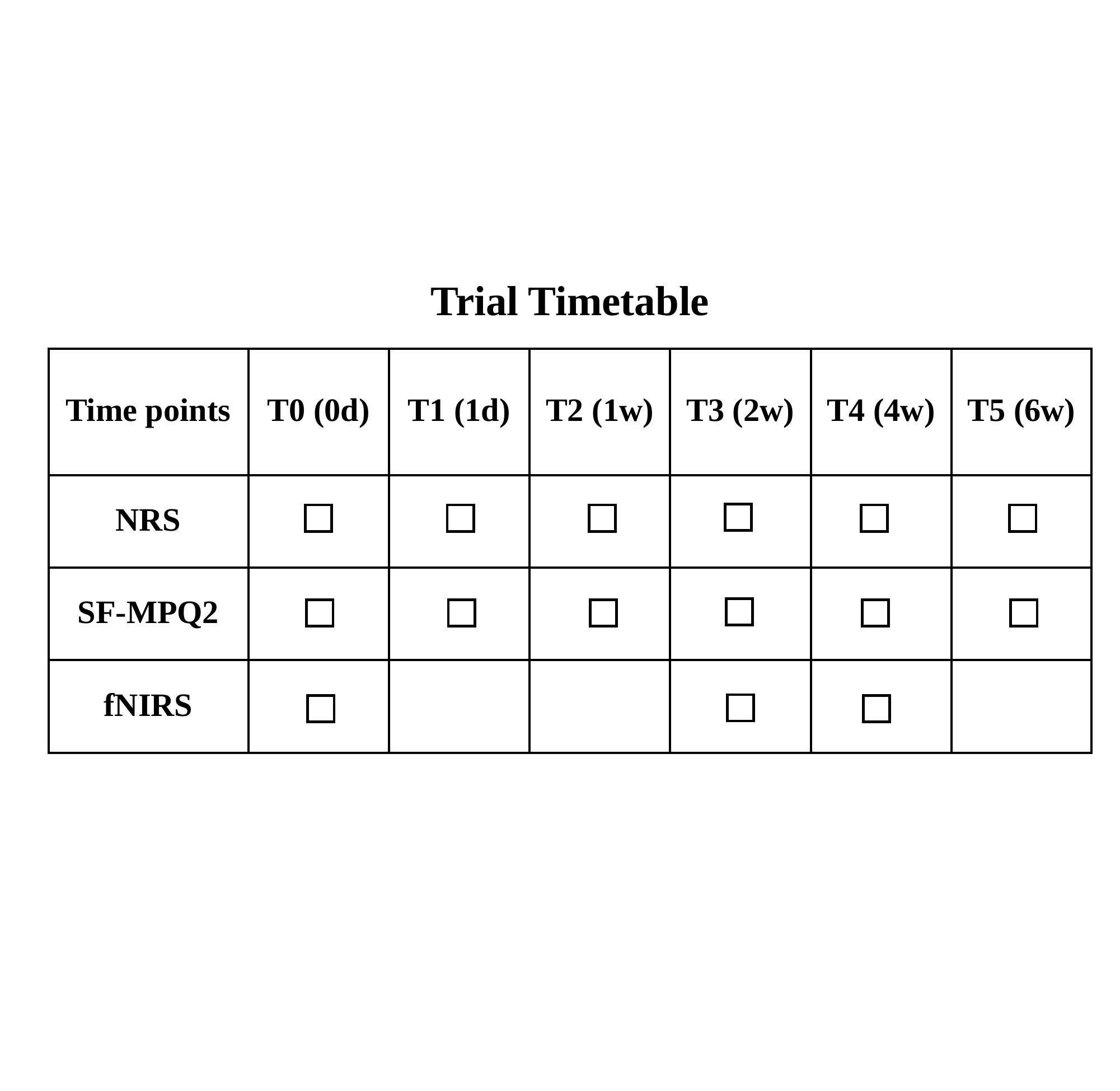

| Trial Timetable | | | | | | |
| --- | --- | --- | --- | --- | --- | --- |
| Time points | T0 (0d) | T1 (1d) | T2 (1w) | T3 (2w) | T4 (4w) | T5 (6w) |
| NRS | | | | | | |
| SF-MPQ2 | | | | | | |
| fNIRS | | | | | | |

Supplement: Supplementary file 3 — Supporting Information 3 Supporting Figure 1 shows the trial timetable. Trial timetable. Pain intensity was evaluated by means of NRS and SF‐MPQ2 at six different time points: T0 (before treatment), T1 (on the first day), T2 (after 1 week), T3 (after 2 weeks), T4 (after 4 weeks), and T5 (after 6 weeks). fNIRS was evaluated only at T0, T3, and T4. Abbreviations: NRS: numerical rating scale; SF‐MPQ2: the Short‐Form McGill Pain Questionnaire‐2; fNIRS: functional near‐infrared spectroscopy. [file PRM-2026-4002703-s003.pptx]

## Slide 1
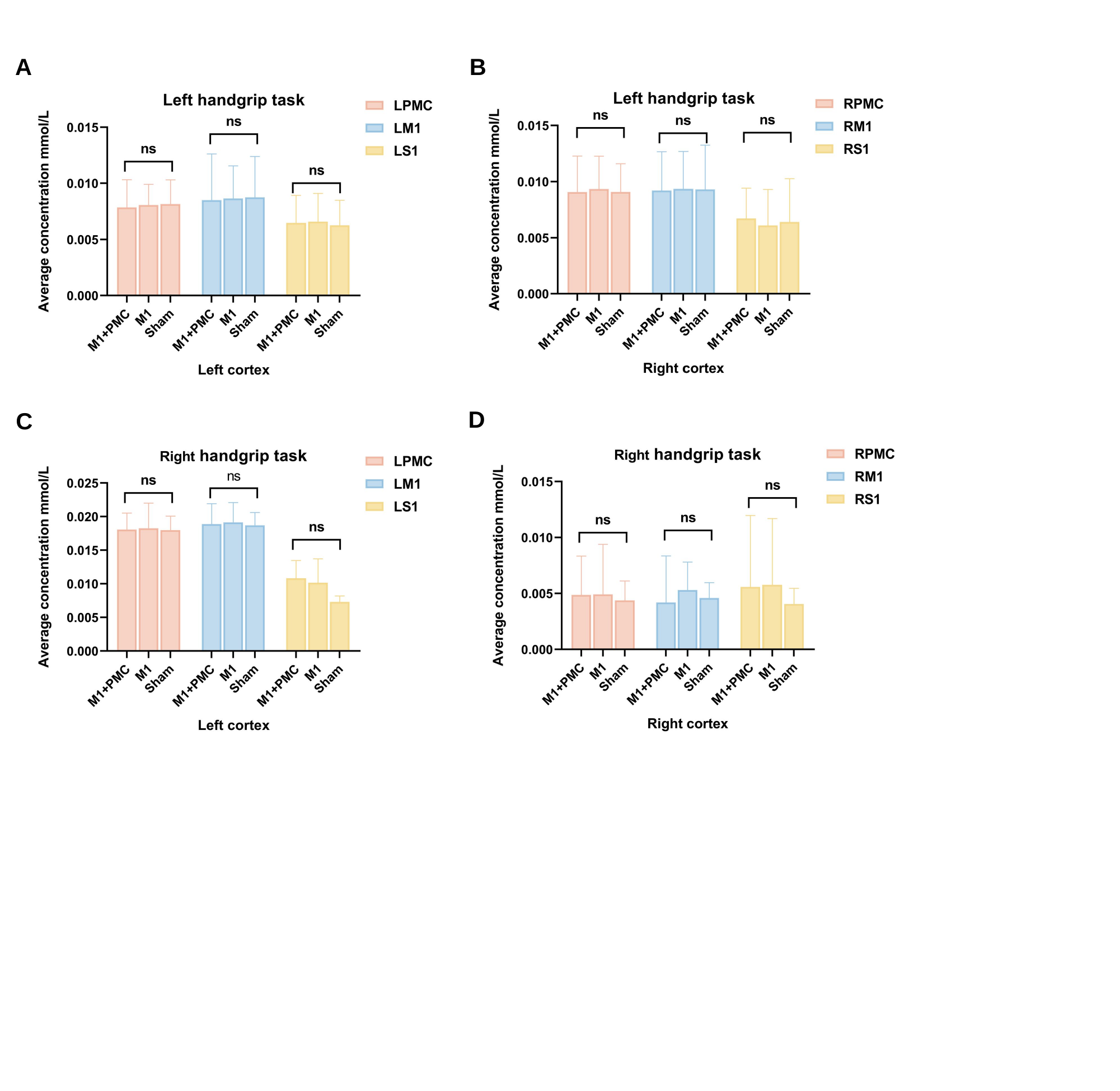

A
B
D
C

Supplement: Supplementary file 4 — Supporting Information 4 Supporting Figure 2 compares the baseline average HbO concentrations in bilateral cortical regions across three groups during both left and right handgrip tasks. (A) Comparison of average HbO concentrations in the left cortex of three groups at baseline for the left handgrip task. (B) Comparison of average HbO concentrations in the right cortex of three groups at baseline for the left handgrip task. (C) Comparison of average HbO concentrations in the left cortex of three groups at baseline for the right handgrip task. (D) Comparison of average HbO concentrations in the right cortex of three groups at baseline for the right handgrip task. Abbreviations: LPMC, left premotor cortex; RPMC, right premotor cortex; LM1, left primary motor cortex; RM1, right primary motor cortex; LS1, left primary somatosensory cortex; RS1, right primary somatosensory cortex. [file PRM-2026-4002703-s004.pptx]
